# Characterizing the Impact of Category Uncertainty on Human Auditory Categorization Behavior

**DOI:** 10.1371/journal.pcbi.1003715

**Published:** 2014-07-17

**Authors:** Adam M. Gifford, Yale E. Cohen, Alan A. Stocker

**Affiliations:** 1 Neuroscience Graduate Group, University of Pennsylvania, Philadelphia, Pennsylvania, United States of America; 2 Departments of Otorhinolaryngology, Neuroscience, and Bioengineering, University of Pennsylvania, Philadelphia, Pennsylvania, United States of America; 3 Departments of Psychology and Electrical and Systems Engineering, University of Pennsylvania, Philadelphia, Pennsylvania, United States of America; Newcastle University Medical School, United Kingdom

## Abstract

Categorization is an important cognitive process. However, the correct categorization of a stimulus is often challenging because categories can have overlapping boundaries. Whereas perceptual categorization has been extensively studied in vision, the analogous phenomenon in audition has yet to be systematically explored. Here, we test whether and how human subjects learn to use category distributions and prior probabilities, as well as whether subjects employ an optimal decision strategy when making auditory-category decisions. We asked subjects to classify the frequency of a tone burst into one of two overlapping, uniform categories according to the perceived tone frequency. We systematically varied the prior probability of presenting a tone burst with a frequency originating from one versus the other category. Most subjects learned these changes in prior probabilities early in testing and used this information to influence categorization. We also measured each subject's frequency-discrimination thresholds (i.e., their sensory uncertainty levels). We tested each subject's average behavior against variations of a Bayesian model that either led to optimal or sub-optimal decision behavior (i.e. probability matching). In both *predicting* and *fitting* each subject's average behavior, we found that probability matching provided a better account of human decision behavior. The model fits confirmed that subjects were able to learn category prior probabilities and approximate forms of the category distributions. Finally, we systematically explored the potential ways that additional noise sources could influence categorization behavior. We found that an optimal decision strategy can produce probability-matching behavior if it utilized non-stationary category distributions and prior probabilities formed over a short stimulus history. Our work extends previous findings into the auditory domain and reformulates the issue of categorization in a manner that can help to interpret the results of previous research within a generative framework.

## Author Summary

Categorization is an important cognitive process that allows us to simplify, extract meaning from, and respond to objects in the sensory environment. However, categorization is complicated because an object can belong to multiple categories. Thus, to inform our categorical judgments, we must make use of prior information. Given the importance of categorization, we hypothesized that humans utilize optimal strategies for making categorical judgments that allow us to minimize categorization errors. We found, though, that whereas subjects used prior information (i.e., category prior probability), they were sub-optimal in their categorization behavior. This seems to be common in other perceptual and cognitive tasks as well. We then explored the bases for this sub-optimal behavior and found that it can be consistent with an optimal strategy if we assume that subjects have trial-by-trial noise in components of the judgment process. This work extends previous similar findings into the field of auditory categorization and provides a means to reinterpret previous results.

## Introduction

Categorization is a natural and adaptive process that allows the brain to organize the typically high-dimensional and continuous sensory information into robust hierarchical and discrete representations. These discrete representations, or categories, are a means to mentally manipulate, reason about, and respond to objects in our environment [1], [2]. For instance, in auditory perception, humans and other animals can ignore the natural acoustic variability that exists between different utterances of the same vocalization in order to differentiate one type of vocalization (e.g., a *howl*) from a second type (e.g., a *bark*). In other situations, listeners can use this variability to identify one caller (e.g., Lassie) from another (e.g., Benji).

The perceptual ease with which we can categorize sound belies the complex computations underlying this ability. One reason categorization is complex is that a sensory property may be ambiguous with respect to the stimulus' category membership. For example, because both dogs and wolves can produce howls, the acoustic structure of the howl by itself may not provide enough information to the listener for proper identification of the caller. In such cases, and in the absence of other sensory information, the listener needs to rely on other sources of information to correctly categorize a sound and identify whether the howl came from a dog or a wolf. This information can be prior knowledge such as knowing that the probability of encountering a wolf is low. Since prior information is subjective, it is of fundamental interest to understand the degree to which an observer acquires this information and then uses it to perform categorical judgments.

The utility of prior information in visual categorization has been well studied [1], [3]–[10]. In comparison, our understanding of how prior information informs categorical judgments in audition is relatively limited and has only more recently become an active area of research [11]–[15]. More importantly, auditory categorization has not been tested or modeled in situations in which the auditory stimulus is ambiguous with regard to its category membership. Understanding auditory-categorization behavior is important for differentiating between modality-specific versus modality-general computational strategies, which can provide insights into the underlying neural computations.

In particular, categorization can be understood as the result of a probabilistic inference process in which the observer combines sensory and prior information according to their relative levels of uncertainty (noise) [16]. Bayesian statistics is a useful mathematical framework to formulate generative models for such categorical inference processes. However, it requires a precise quantification of the different levels of uncertainty in order to provide behavioral predictions that allow for unique model interpretations. For example, different decision strategies can lead to very similar model predictions if the sensory noise levels are allowed to be free parameters.

The purpose of this study was two-fold: (1) to test whether human subjects can learn and use category-prior information when making auditory categorical judgments and (2) to carefully constrain and validate a generative Bayesian model of auditory categorization against experimental data. To this end, we developed a novel auditory categorization task that required subjects to categorize the frequency of a tone burst into one of two overlapping categories (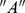 or 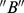). We systematically varied the prior probability of choosing a frequency from category 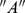 or 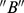 in different blocks of the experiment. Furthermore, we determined each subject's sensory uncertainty by measuring individual frequency-discrimination thresholds. Based on these uncertainty measurements, we formulated a Bayesian model to individually quantify how well each subject learned the categorical priors (i.e., the category distributions and prior probabilities) and to test whether subject's employed an optimal decision strategy. We found that most subjects appropriately learned the different category prior probabilities, yet showed some variability and uncertainty in the shape of the learned category distributions. Furthermore, given the measured sensory uncertainty during the experiment, subjects' overall behavior was more consistent with probability matching rather than an optimal decision strategy for category choice. Further analyses indicated that overall probability-matching behavior could emerge if, trial-by-trial, subjects employed an optimal decision strategy and assumed non-stationary categorical priors.

## Methods

### Ethics statement

All subjects participated in a purely voluntary manner, after providing informed written consent, under the protocols approved by the Institutional Review Board of the University of Pennsylvania.

### Experimental setup

Six subjects (two female) participated in two tasks: (1) a discrimination task that estimated each subject's frequency-discrimination thresholds and (2) an auditory-categorization task that tested how each subject used category-prior information. Both tasks were conducted in a darkened anechoic chamber (2 m×1.5 m, Industrial Acoustics Company, Inc.), which housed a chair for the subject, a gamepad, a table mounted with an LCD computer screen (P190S, Dell, Inc.), a speaker (MSP7, Yamaha, Inc.), and a chin rest. The speaker was positioned ∼0.1 m below a subject's ears when his/her head was placed on the chin rest. The gamepad registered the subject's responses during each task. Both the discrimination and categorization tasks were designed and implemented in MATLAB (version R2010b) with the Tower-of-Psych and Snow-Dots packages (freely available resources [17], [18]). For both tasks, the stimuli were 750-ms tone bursts (10-ms 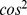 ramp; frequency range: 500–5550 Hz). The tone frequencies were distributed uniformly in 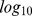 units. Stimuli were synthesized with an RX6 Multifunction Processor (Tucker-Davis Technologies, Inc.) with a sampling rate of 25 kHz and were presented at 65 (± 3) dB SPL.

### Discrimination task and analysis

Each subject participated in a two-interval, two-alternative forced choice frequency-discrimination task. This task measured each subject's frequency-discrimination threshold at eight different “standard” frequencies, which were distributed between 500–5550 Hz: 794, 1260, 2297, 2639, 3031, 3482, 4462, and 4976 Hz. A trial began with a visual “GO” cue on the computer screen, followed by the presentation of the first tone burst. After a 1000-ms delay, the second tone burst was presented. Following offset of this second tone burst, the subject had 2000 ms to report which tone burst had the higher frequency. Subjects only received feedback (in the form of a yellow circle on the computer screen) when a response was not made within the allotted response window.

In each trial, one tone burst was one of the standard frequencies, whereas the other “comparison” tone burst had a different frequency. We used a 2-up-1-down adaptive staircase procedure [19] to adjust the frequency of the comparison tone across trials. On a trial-by-trial basis, the order of the standard and comparison tone bursts was randomized, as well as the choice of the standard tone burst. Each subject participated in 2–4 experimental sessions. Each session consisted of two blocks of trials; each block contained 30 or 40 trials per standard tone frequency (320 or 480 total trials).

The data for each subject were collapsed across sessions and only trials in which a response was made within the allotted response window were included in subsequent analyses. We computed a psychometric function representing the probability that the subject reported the comparison tone (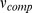) as higher than the standard tone (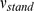). Since the values of 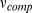 varied across subject and session, 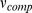 values were binned into five equidistant bins (in 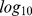 units) for each 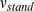 and subject. Each subject's psychometric functions (i.e., one function for each standard tone frequency) were fit with a cumulative Gaussian with free parameters 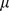 and 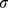 using a maximum-likelihood fitting procedure to the raw data.

We assumed that a subject's discrimination process was the result of a comparison between the frequencies of the standard and comparison tone bursts. We also assumed that the subject's sensory measurements of the comparison and standard tone bursts followed Gaussian distributions, each with the same standard deviation, 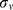, that we defined as the frequency-discrimination threshold of that standard tone frequency 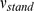 [20]–[22]. Consequently, 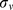 was calculated directly from the 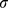 derived from the cumulative Gaussian fit: 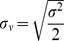. We then computed each subject's frequency-discrimination threshold as the average of the values measured at each of the eight standard tone frequencies (in 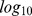 units). We used this average value for the predictions of our Bayesian model (see **Bayesian model**).

### Categorization task and analysis

Each subject then participated in a two-alternative, forced-choice categorization task. The subject reported whether the frequency of a tone burst was a member of one of two different frequency categories (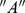 or 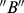).

**Figure 1:**
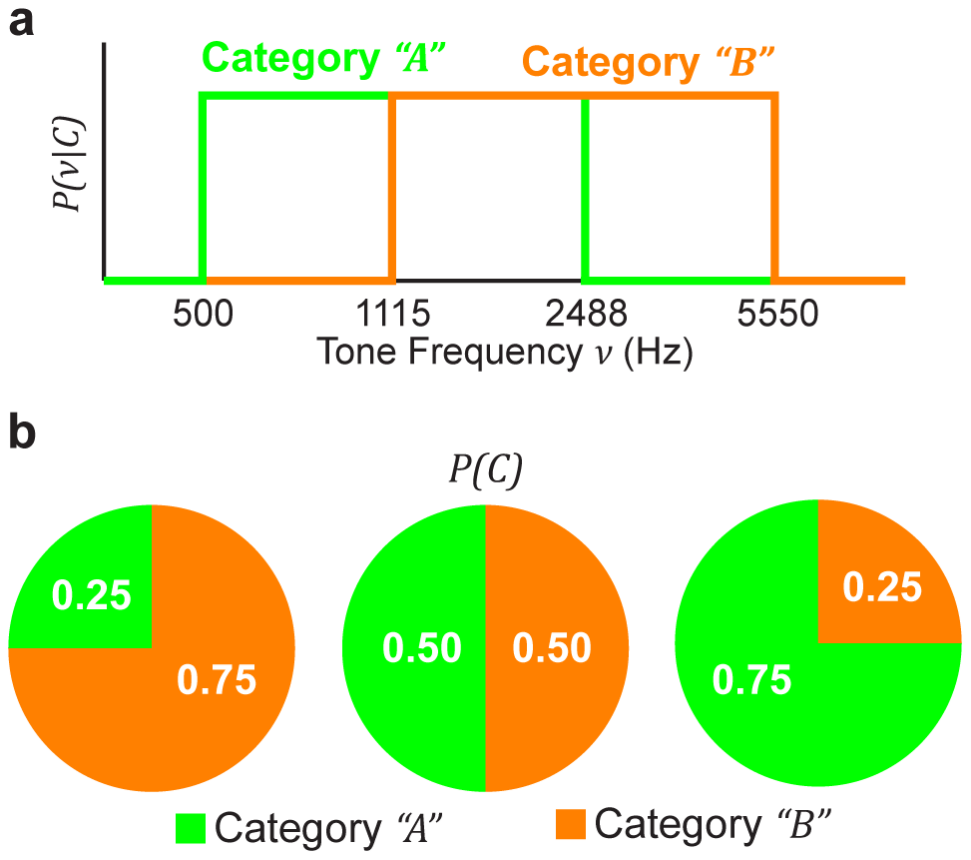
Schematic diagram of the categorical priors employed in the categorization task. (a) The category distributions over tone-burst frequency are piecewise uniform, such that all frequencies for a particular category are equally likely. (b) Three category prior probabilities were employed in separate blocks of trials by varying the proportion of trials that presented a tone belonging to each category. Here, 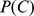 represents the category prior probability, where 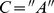 or 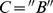.

The frequency range between 550–5550 Hz was divided into two equal (in 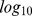 units), but overlapping, piecewise-uniform category distributions (Figure 1a). Category 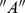 contained frequency values between 500 to 2488 Hz. Category 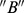 contained frequency values between 1115 to 5550 Hz. These two categories were designed so that category 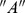 comprised the *lower* two-thirds of the frequency range, whereas category 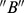 comprised the *upper* two-thirds of the frequency range (again in 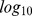 units). As a consequence of this design, one part of each category's distribution was exclusive to that category (i.e., the extreme thirds of the entire frequency range), whereas the other part was shared with the other category (i.e., the middle third of the range).

Our critical experimental manipulation was to vary the category prior probabilities, 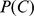, where 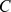 was either category 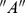 or category 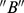. We varied the prior probabilities, on a block-by-block basis, by appropriately selecting the proportion of trials originating from a particular category. We tested the influence of three different category prior probabilities (Figure 1b). In two of the manipulations, it was more likely that the frequency of a tone burst originated from one category than the other. In the third manipulation, it was equally likely that the frequency of a tone burst originated from either category.

Before the first session, the category prior probabilities were explained to each subject. A trial began with a brief 1500-ms countdown, followed by a visual ‘GO’ cue indicating the imminent presentation of a tone burst. After tone-burst offset, the subject had 1000 ms to report a choice. Subjects received visual feedback on every trial: a green circle for correct responses, a red circle for incorrect responses, and a yellow circle for no response within the allotted 1000-ms response window. In separate blocks of trials, the prior probability for category 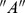 was one of three values: 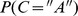 = 0.25, 0.5, or 0.75. On a trial-by-trial basis, we randomly selected the category according to its prior probability. Once a category was selected, we randomly selected a frequency from that category. As noted above, because the category distributions were piecewise uniform, any stimulus within the category was equally likely: 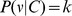 for all frequencies 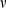 within the category distribution (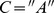 or 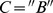) and 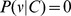 outside of the distribution. The value of 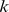, where 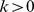, is defined by the width of the category distributions.

Each subject participated in 3–5 sessions of the categorization task; each session included one block of each of the three category prior probabilities. In total, each subject completed between 600–1000 trials for each category prior probability.

For each subject, we computed the psychometric function 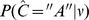 (where 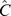 represents the subject's category choice) for each of the three category prior probabilities across all sessions. Tone frequencies were binned into nine equidistant bins that spanned the entire frequency range: three frequency bins in each of the two unambiguous frequency regions and three bins in the ambiguous frequency region. We fit each psychometric function with a cumulative Gaussian using a maximum-likelihood procedure and identified the frequency at which a subject was equally likely to choose 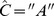 or 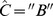: that is, the point of subjective equality (PSE). We also fit cumulative Gaussians to each subject's categorization performance separately for each session to test for any potential learning effects throughout the course of the experiment.

### Bayesian model

We developed a Bayesian model that tested three key aspects of each subject's categorization behavior. First, we tested whether subjects used the category-prior information for their categorical decisions. Second, we tested the degree to which subjects were able to learn category distributions. Finally, we tested the degree to which subjects employed an optimal decision strategy given the characteristics of the categorization experiment.

**Figure 2:**
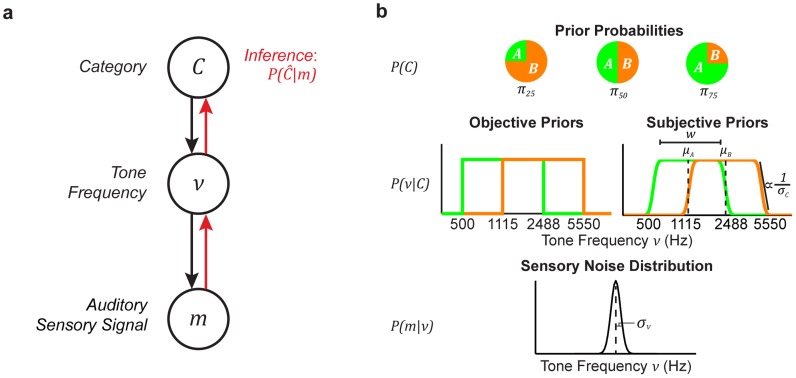
Graph of the Bayesian model. (a) The category identity 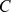 of the frequency of a tone burst (top level) constrains the values of the tone frequency 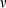 (middle level). The auditory sensory signal 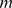 represents a noisy measurement of the true tone frequency 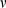. The black arrows define the generative conditional probability densities 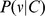 and 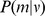, respectively. The task of the observer is to infer the category membership of the tone's frequency from this noisy sensory measurement 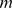 (red line from bottom to top level). (b) The category identity is modeled probabilistically using three 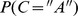 conditions in the categorization task (top panel). Given a particular category, the probability of a certain tone frequency is governed by the respective conditional distribution for frequency 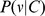 (middle panel). The sensory process of the Bayesian observer is modeled as a Gaussian process centered at the true stimulus frequency (bottom level). The width 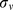 reflects the degree of uncertainty in the sensory process due to noise and determines an observer's ability to discriminate tones of different frequencies. Thus, we constrained this width with data from an additional discrimination experiment.

Categorization can be considered an inference process over the generative graphical model shown in Figure 2a. The true category 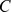 of a stimulus is governed probabilistically according to the prior probability 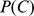 (Figure 2b, top panel). The category distribution, 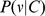, indicates the probability that a stimulus from a category 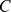 has a certain tone frequency 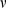. We assumed that each tone with frequency 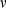 generated a sensory signal 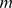 according to the probability density 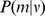, which characterized the sensory uncertainty and noise in the auditory pathway. We assumed 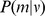 to be Gaussian with a mean centered on the true tone frequency 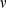 and a standard deviation 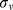 that reflected the level of sensory uncertainty (Figure 2b, bottom panel). We measured 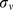 for each subject as his or her frequency-discrimination threshold (see **Discrimination task and analysis**).

We assumed that subjects performed Bayesian inference over this generative model when solving the categorization task: given the sensory evidence 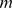, subjects computed the posterior probability 
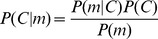
. In this equation, 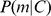 is the likelihood that the measured frequency belonged to a particular category 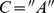 or 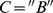. The likelihood 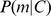 was calculated by marginalizing over the tone frequency as 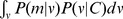. We assumed that subjects either (1) learned the experiment's stimulus distributions (“objective priors”; Figure 2b, middle-left) or (2) only learned an approximation of these distributions (“subjective priors”). For the latter case, we parameterized 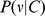 using two piecewise-uniform distributions, each convolved with a Gaussian (Figure 2b, middle-right). The subjective category distributions can be thought of as noisy estimates of the objective distributions. Each subjective distribution had its own mean (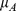 and 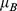) but had the same distribution width (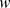) and the same Gaussian standard deviation (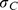). Finally, similar to the category distributions, the values of the category prior probability 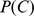 were assumed either to be (1) the experimental prior probabilities (objective priors) or (2) the free parameters 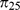, 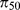, and 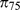, representing each category prior probability (subjective priors).

Based upon the posterior 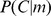, we tested whether subjects employed an optimal decision strategy to make a category choice (either 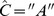 or 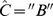). This strategy is a *maximum a posteriori* (MAP) strategy, in which subjects chose the most probable category given 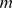. In other words: 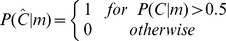. Thus, the subjects chose 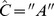 if 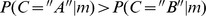, and chose 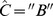 otherwise.

We also tested whether subjects' decisions reflected probability matching (MATCH) as a general index of sub-optimal categorization behavior [23]–[25]. Probability matching is equivalent to a decision strategy that results in subjects choosing a category probabilistically according to the posterior probability 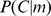. In other words, 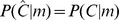.

Finally, to directly compare and fit the model's predictions to each subject's behavioral data, we computed the psychometric function as a function of the true frequency 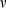 as 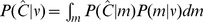.

### Model predictions and fits

Assuming objective priors, we used the Bayesian model to *quantitatively predict* each subject's categorization performance. We assumed the likelihood function 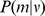 was a Gaussian distribution with a standard deviation 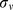, which was measured and fixed separately for each subject (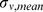; see **Discrimination task and analysis**). Under these assumptions, the model has no free parameters. Therefore, we could *predict* each subject's psychometric function for each category prior probability and for both optimal (MAP) and sub-optimal (MATCH) categorization. We calculated the quality of the MAP and MATCH predictions by computing their respective log-likelihood values across all 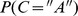 conditions. We rescaled these log-likelihood values relative to the predictions of two reference models: (1) an empirical model, which represents how well the observed data explains itself (i.e., a binomial model that employs the empirical choice probabilities), and (2) a random-guessing model [26].

Assuming that subjects only learned noisy estimates of the categorical priors (i.e., subjective priors), we also computed maximum-likelihood fits of the model for both MAP and MATCH behavior to each subject's categorization performance. The sensory uncertainty 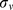 was again *fixed* for each subject based on the results of the discrimination experiment. Thus, the model fit with the subjective priors had seven free parameters, namely 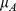, 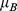, 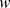, 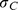, 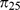, 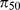, and 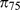 (see Figure 2b and previous section). We tested the goodness of fits by again comparing the normalized total log likelihoods for both MAP and MATCH.

Finally, to assess the full potential of either type of decision behavior to explain each subject's categorization performance, we computed maximum-likelihood fits of the model using subjective priors, this time including 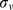 as an additional free parameter (for a total of eight free parameters). Once again, we tested the goodness of fits by comparing the normalized total log likelihoods.

## Results

### Individual subject's frequency-discrimination thresholds

We measured each subject's frequency-discrimination threshold to determine individual sensory uncertainty. The frequency-discrimination experiment required subjects to indicate the interval that contained the higher-frequency tone burst.

**Figure 3:**
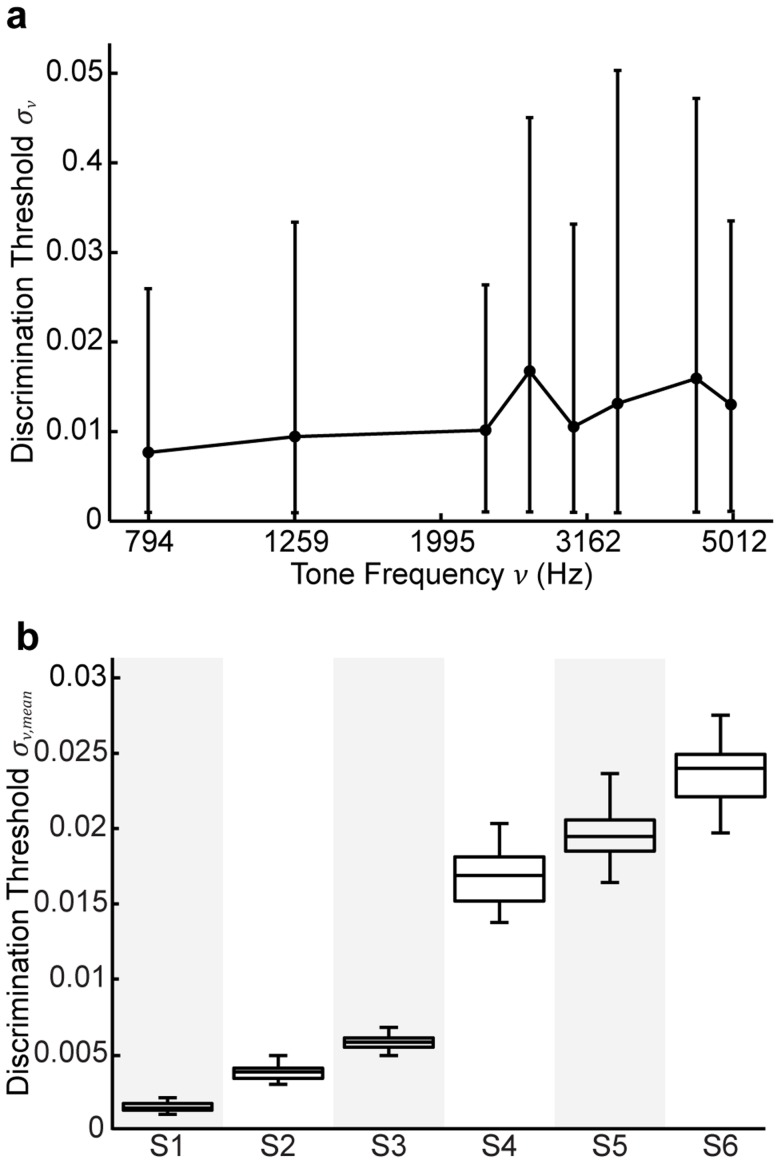
The discrimination thresholds for each subject. (a) Mean discrimination thresholds and 95% confidence intervals (CIs) as a function of standard frequency 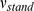 across subjects. The discrimination thresholds were derived from the widths of the cumulative Gaussian fits to each subject's psychometric function for frequency discrimination at each 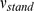. (b) Overall discrimination thresholds across standard frequencies for each subject, computed as the mean across all 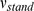 values. Boxplots denote the bootstrapped median, 50%, and 95% CIs of the overall discrimination threshold. The subjects are ordered by increasing median of the overall discrimination threshold, 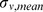.

For each subject, we calculated discrimination thresholds 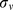 for each standard frequency, which is summarized in Figure 3a. As expected [27]–[30], we found that the thresholds were approximately constant across the tested frequency range. Consequently, for each subject, we computed the mean of the thresholds (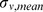) across the eight standard frequencies (Figure 3b). We used 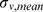 as the measure of each subject's sensory uncertainty in our Bayesian model.

### Human subjects can quickly learn category priors

**Figure 4:**
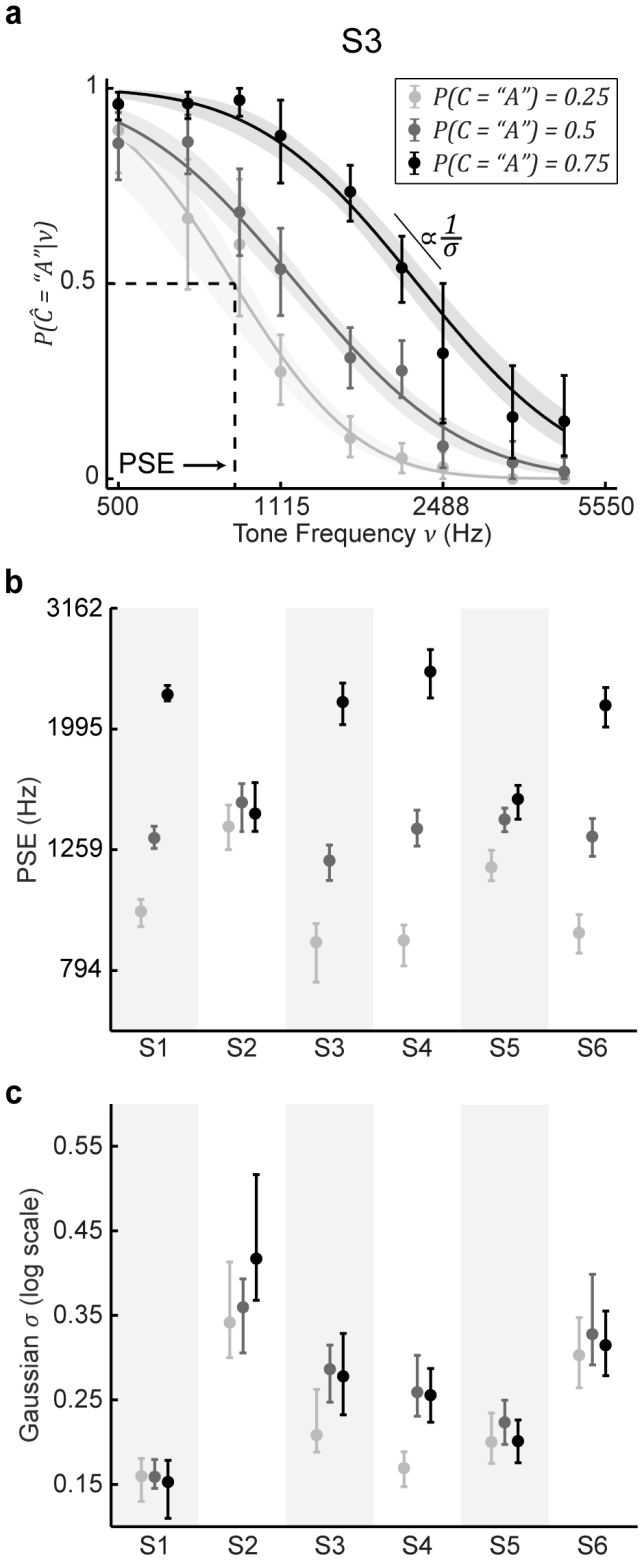
Effects of category priors on psychometric data for individual subjects. (a) Psychometric functions depicting the probability of choosing 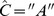, given the true tone frequency, for an example subject. Data points denote observed performance calculated by binning stimulus frequencies into nine equidistant bins. Lines depict cumulative Gaussian fits to raw data. Shading of lines and data points denote 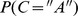 condition. Error bars and shaded regions represent bootstrapped 95% CIs. (b) Medians and bootstrapped 95% CIs of the PSE of the fitted psychometric functions for each prior probability and subject. (c) Medians and bootstrapped 95% CIs of the 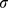 values of the fitted psychometric functions for each prior probability and subject. The 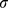 values are plotted in 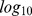 units. For (b) and (c), shading of the data points denotes the different 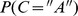 conditions.

Because the subjects were initially unaware of the categorical priors, subjects had to learn both the category distributions and the category prior probabilities to make informed category decisions. To test whether subjects learned this information, we first compared each subject's psychometric functions (i.e., 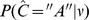) across the three different values of the category prior probability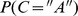. We fit these psychometric functions with a cumulative Gaussian and extracted the point of subjective equality (PSE) for each curve. The psychometric functions and Gaussian fits for an example subject (S3) are depicted in Figure 4a. Two main points can be taken from this figure. First, as the tone frequency increased, the probability that the subject chose 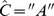 decreased. Second, as 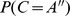 increased, the psychometric functions shifted toward higher tone frequencies. However, the slopes of the psychometric functions remained consistent across category prior probability. These effects were comparable across individual subjects, with all but subject S2 exhibiting clear effects of the different category prior probabilities. These findings are summarized in Figure 4b and 4c.

**Figure 5:**
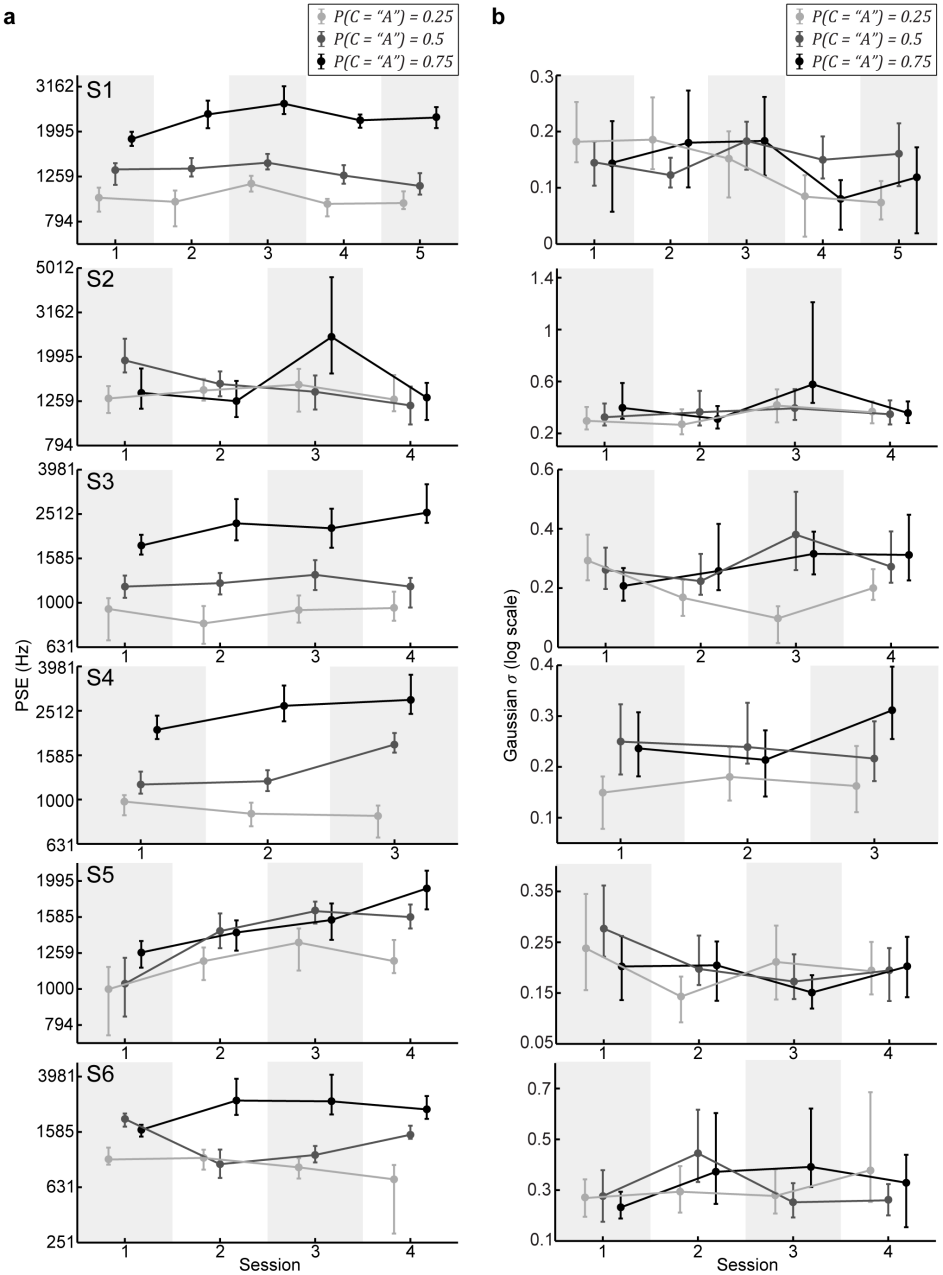
Effects of learning. The extracted PSEs (a) and slopes (b) for each subject as a function of session. For both sets of plots, the data points represent the median and 95% CIs based on bootstrapped behavioral data. Shading denotes the different prior probabilities.

These effects of the different category prior probabilities were evident as early as the first session. Generally, additional experience with the categorical priors had little differential effect on PSE and slope (Figure 5). Thus, for subsequent analyses we grouped each subject's data across sessions.

### Bayesian-model predictions

**Figure 6:**
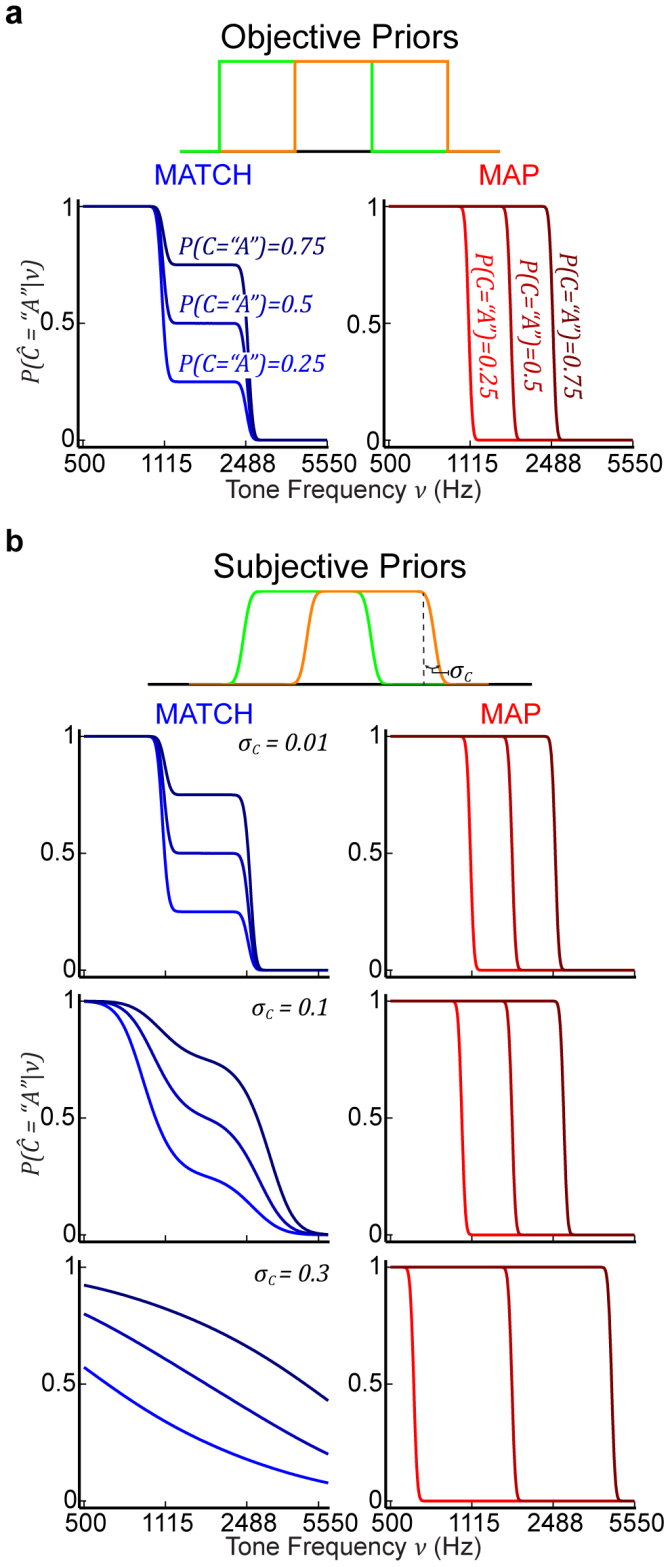
Predictions of the Bayesian model with different categorization behaviors. (a) Predicted psychometric functions for the model with objective priors during each of the three prior-probability conditions. The predictions assuming probability-matching (MATCH) behavior are on the left, whereas those of the MAP decision strategy are on the right. (b) Predicted psychometric functions for the model with subjective priors. Example predictions are plotted for three selected values of 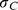 for MATCH (left) and MAP (right). Line colors distinguish MAP versus MATCH and color shade denotes the three prior probabilities. For all model predictions, 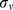 was fixed to the mean discrimination threshold across all subjects.

Our Bayesian model makes distinct predictions for subjects' psychometric performance (Figure 6). In the lowest and highest thirds of the frequency range, choice behavior is independent of category prior probability and identical for MATCH and MAP. This independence occurs because these frequency ranges are exclusive to categories 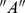 and 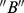, respectively. The effects of 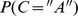 are only present in the middle third of the frequency range, where the category distributions overlap. Under the objective-priors assumption, probability matching (Figure 6a, left) yields psychometric functions that exhibit a characteristic plateau. Increasing 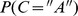 causes *vertical shifts* in these plateaus. In contrast, the MAP decision strategy (Figure 6a, right) yields smooth, sigmoidal psychometric functions. Moreover, increasing 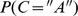 causes *lateral shifts* of the psychometric function. For both behaviors, 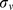 governs the steepness of the transition in choice behavior from choosing 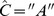 to choosing 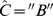.

Under the subjective-priors assumption, the predicted characteristics of the psychometric functions change distinctly for MAP and MATCH (Figure 6b). With MATCH, the psychometric functions become smoother overall with increasing values of 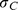 (Figure 6b, left column). However, the vertical shifts with increasing 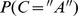 are still evident. The predictions for the MAP decision strategy are similar to those under the objective-priors assumption (compare Figures 6a and 6b, right column). Contrary to what is seen in the predictions for MATCH behavior, here 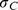 does not affect the slopes but, instead, affects the relative lateral shifts of the psychometric functions.

### Data versus model predictions for objective priors

**Figure 7:**
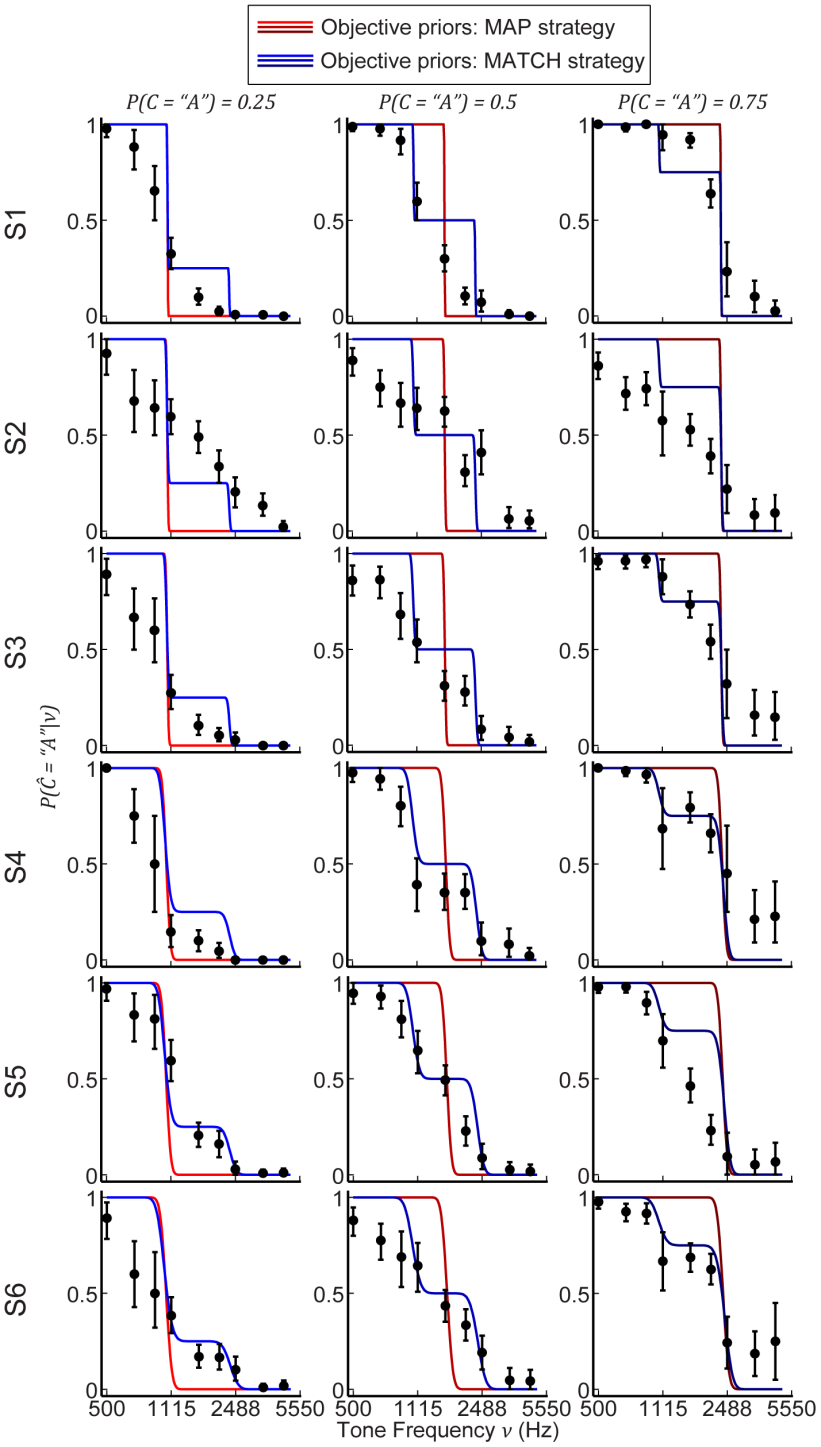
Model comparisons using objective priors and individually measured sensory noise 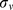. Rows distinguish the responses from and predictions for each subject. Columns distinguish the three prior probabilities. For all plots, the data points represent mean performance and bootstrapped 95% CIs in the categorization task. Line colors distinguish MAP versus MATCH and color shade denotes the three prior probabilities.

We compared the predictions of the Bayesian observer with each subject's behavior assuming the objective priors (see **METHODS**). In general, the model predictions for both types of decision behavior did not accurately reflect subjects' behavior (Figure 7). MATCH behavior predicted step-like psychometric functions (see Figure 6) that were reflected only in some subjects' performance (e.g. S4). The predictions of the model with the MAP decision strategy were even less accurate: this decision strategy predicted slopes of the psychometric functions that were substantially and consistently steeper than those observed in each subject.

**Figure 8:**
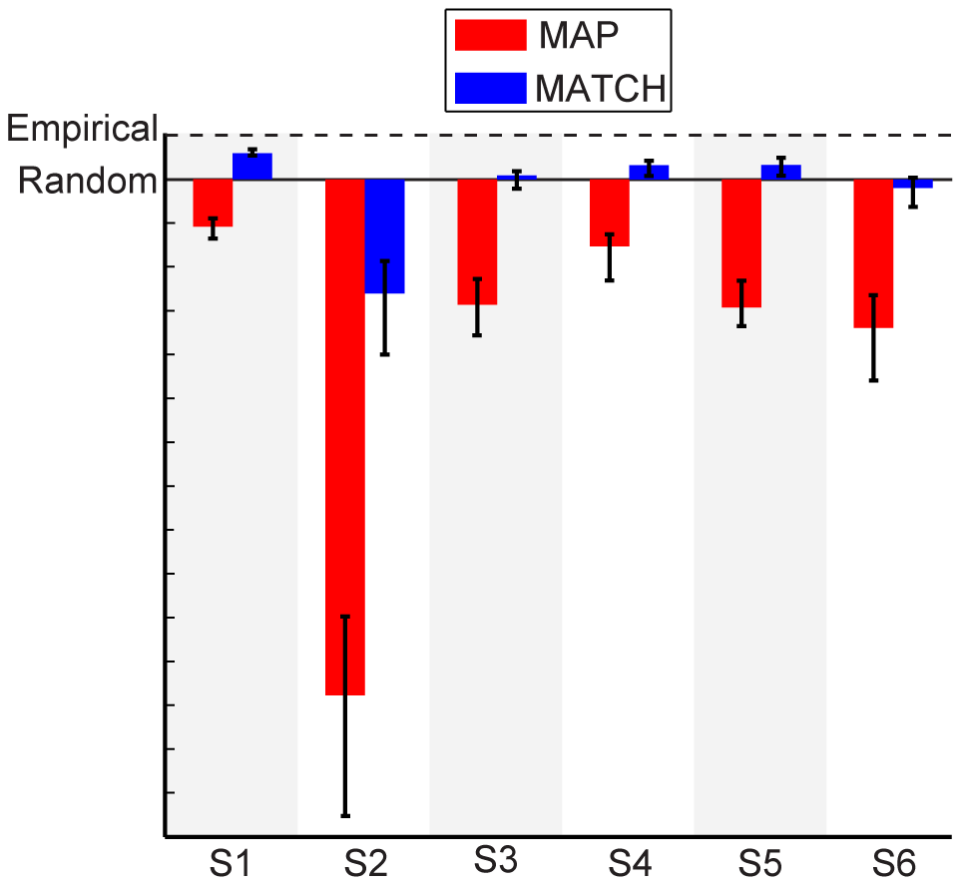
Normalized likelihoods for the Bayesian model predictions. Likelihoods are normalized between that of a random-guessing model and empirical performance, defined as how likely the measured performance explains itself (see METHODS). Color denotes MAP versus MATCH.

We quantified the quality of the two model predictions by calculating the total likelihood of the models given each subject's behavior. MATCH was significantly more predictive of each subject's performance, as exemplified by the likelihoods for each type of decision behavior across subjects (Figure 8). In fact, the MAP strategy was significantly worse than a random guess for all subjects, whereas MATCH was better than random guessing for half of the subjects (i.e., S1, S4, and S5).

### Data versus model fits with subjective priors

Because the objective category distributions did not fully predict the subjects' performances, we used subjective categorical priors and fit the Bayesian model (see Figure 2 and **METHODS**). However, as before, we fixed 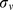 to reflect each subject's measured frequency-discrimination threshold.

**Figure 9:**
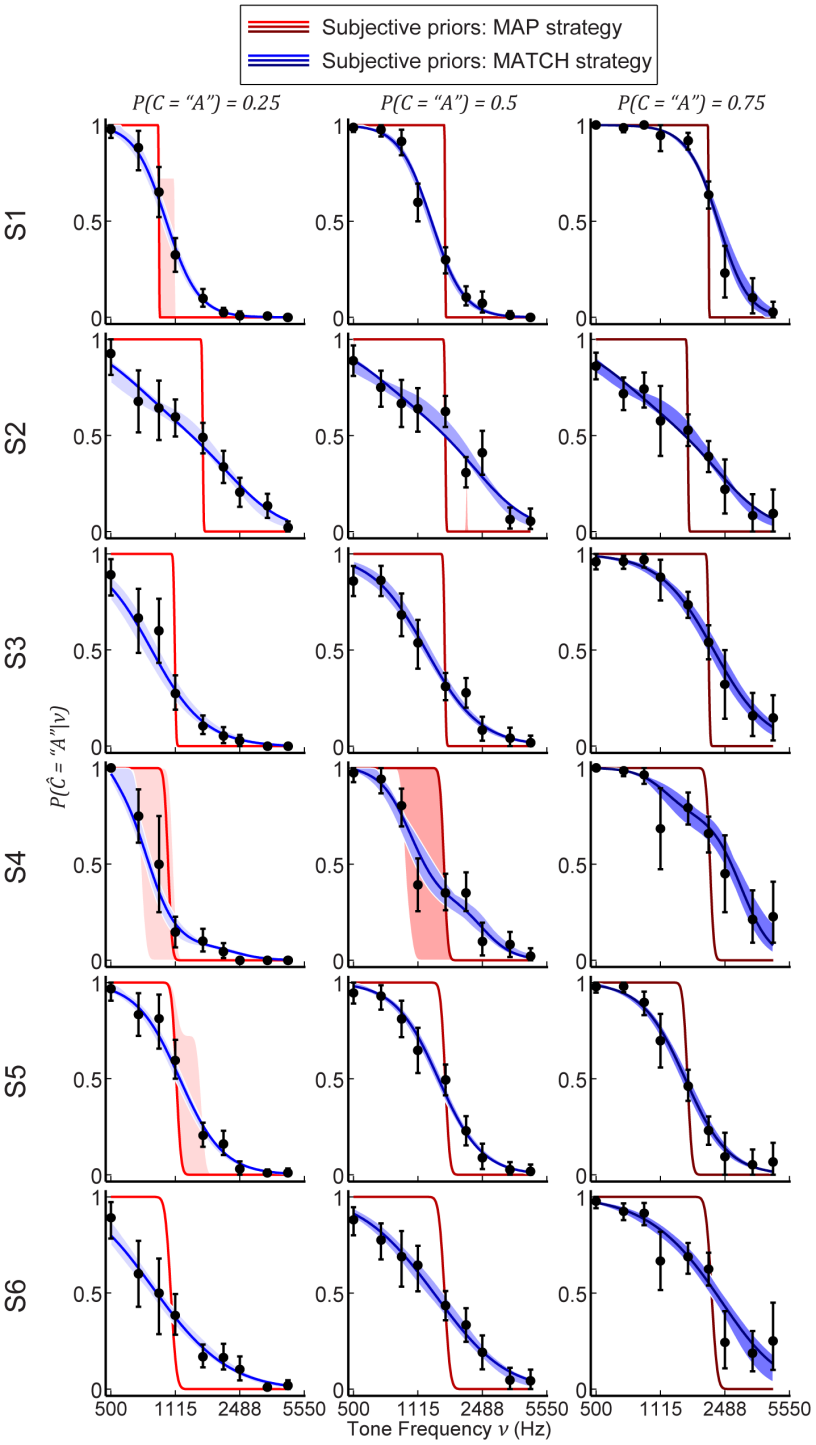
Model comparisons using subjective prior distributions with observed individual responses. The format of the data is the same as that in Figure 7. For all plots, shaded regions denote bootstrapped 95% CIs for subjective-prior model fits.

**Figure 10:**
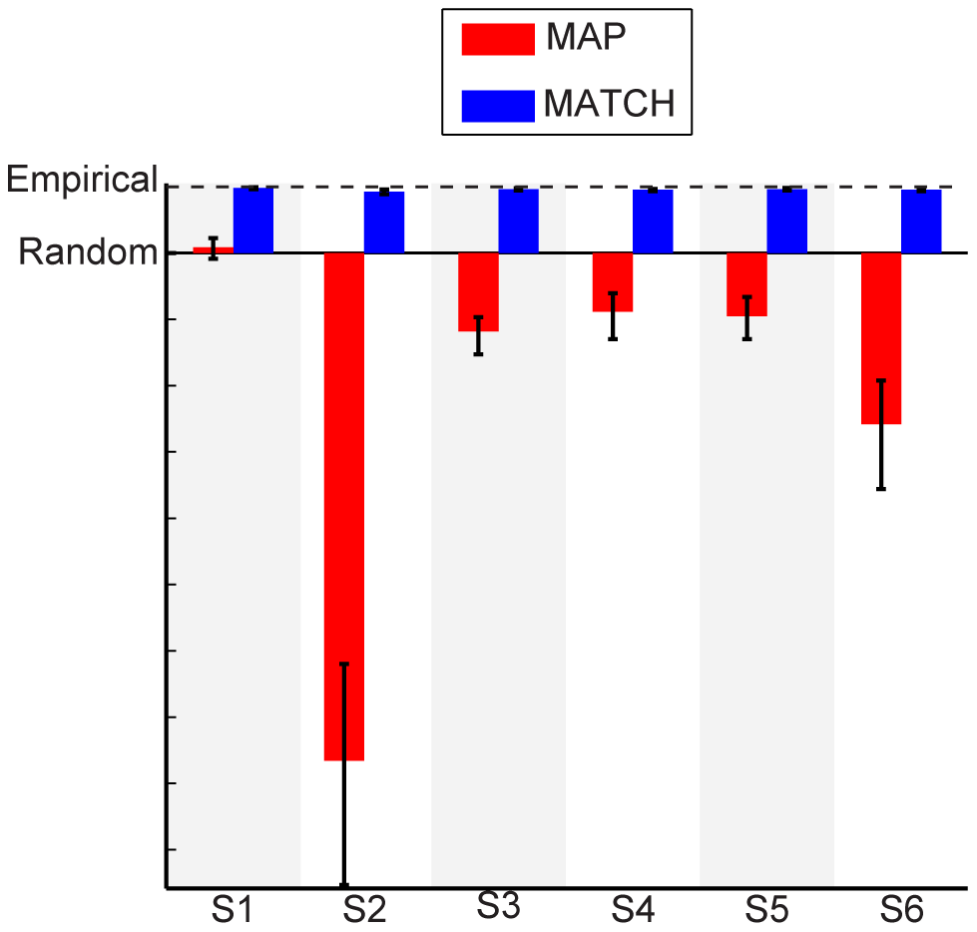
Normalized likelihoods for the Bayesian-model fits assuming subjective priors. The format of the data is the same as that in Figure 8.

Fits assuming MATCH behavior almost perfectly accounted for the data, with an accuracy that approached empirical performance (Figures 9 and 10). However, the fits under the MAP strategy were still poor: the MAP strategy failed to account for the slopes of the psychometric functions (Figure 9). Except for subject S1, the MAP strategy yielded fits that were significantly worse than random guessing. In fact, the MAP-strategy fits to the data did not provide any better account of the data than its predictions based on the objective priors (compare Figures 8 and 10).

### Subjective category distributions and prior probabilities

Finally, we were interested in reconstructing the subjective category distributions for the subjects and comparing them to the objective distributions; because the MAP decision strategy provided a poor description of subjects' performances, we focused only on the fits assuming MATCH behavior.

**Figure 11:**
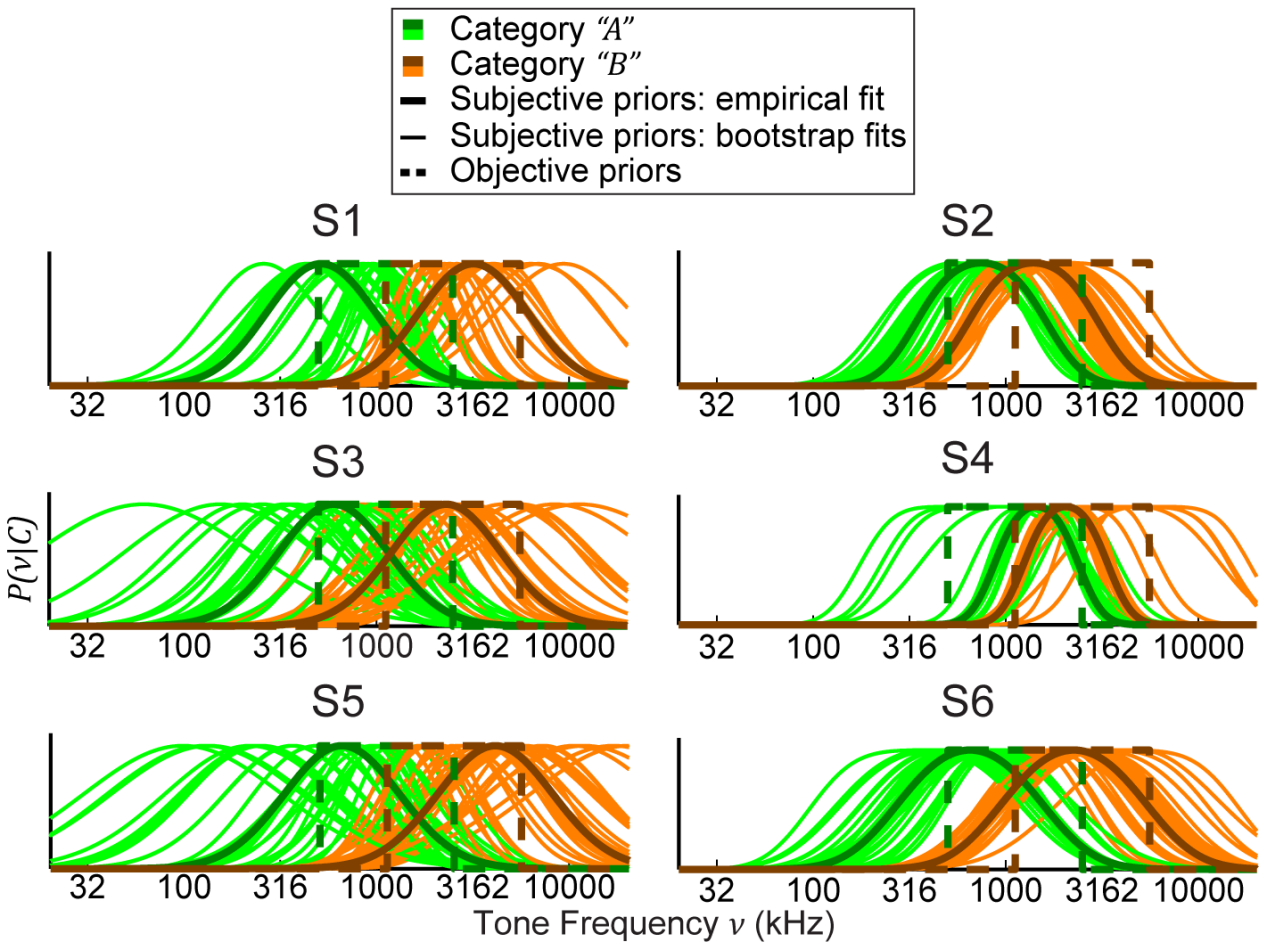
Individual subjects' reconstructed category distributions from the model fits for MATCH behavior. Green traces denote distributions for category 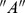, whereas orange traces denote distributions for category 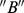. Thick solid lines denote the category distributions calculated from the fit to each subject's observed performance. Thin solid lines denote category distributions from the individual bootstrap fits. Thick dashed lines denote objective priors for comparison.

**Figure 12:**
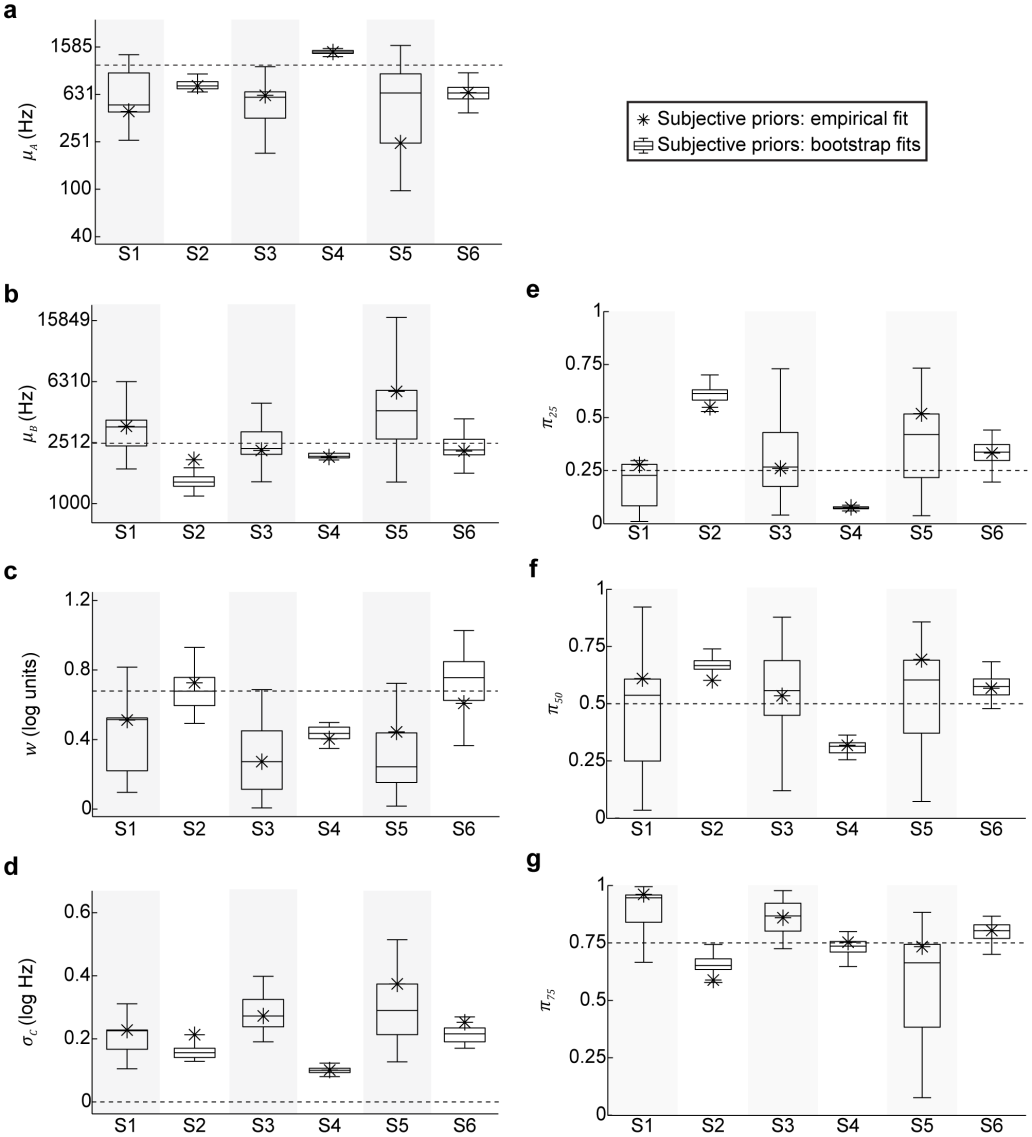
Fitted model parameters for MATCH behavior of the model with subjective priors. (a–d) Boxplots depicting the range of (a) the fitted means for the category-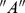 distribution; (b) the fitted means for the category-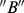 distribution; (c) the fitted widths that were shared between both category distributions; and (d) the widths of the fitted Gaussian functions that were convolved with the fitted uniform distributions. For plots (a–d), the thin dashed lines denote depict the values of 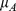 (a), 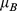 (b), 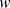 (c), and 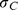 (d) that reflect the objective priors. (e–g) Boxplots depicting ranges of the fitted prior probability parameters 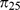, 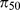, and 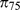. Thin dashed lines denote experimental prior probability values. For all plots, the stars denote values of parameters fit to the measured data, whereas the boxplots denote the median, 50%, and 95% CIs of the parameter values estimated from bootstrapped empirical responses. Note that subject S2's categorization performance was not influenced by the category prior probabilities.

The reconstructed category distributions tended to more closely resemble Gaussian distributions rather than boxes (Figure 11). Both the modeled category means and category widths either were close to or overlapping with the actual means and widths of the objective distributions (Figure 12a–c). However, the category edges were much less defined as compared to the edges of the objective distributions, exemplified by large 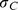 values (Figure 12d). Overall, the fitted category prior probabilities 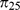, 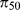, and 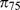 for individual subjects were remarkably similar to the actual values 0.25, 0.5, and 0.75, respectively (Figure 12e–g).

### Analysis of categorization behavior with subjective priors and all free parameters

The previous model analyses revealed that probability matching (MATCH) is much better than the optimal (MAP) strategy in both *predicting* each subject's categorization behavior as well as explaining behavior after *fitting* the model with subjective priors. However, this comparison assumes that we have accurately measured each subject's sensory uncertainty. It is possible that, with additional sources of sensory uncertainty (e.g., memory noise [31], [32]), the MAP strategy could be equally as descriptive as MATCH behavior. Indeed, under certain noise conditions, MAP and MATCH behaviors are equivalent [33]. To address this possibility, we performed an additional analysis in which all of the parameters were fit, including 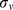 (for a total of eight free parameters).

**Figure 13:**
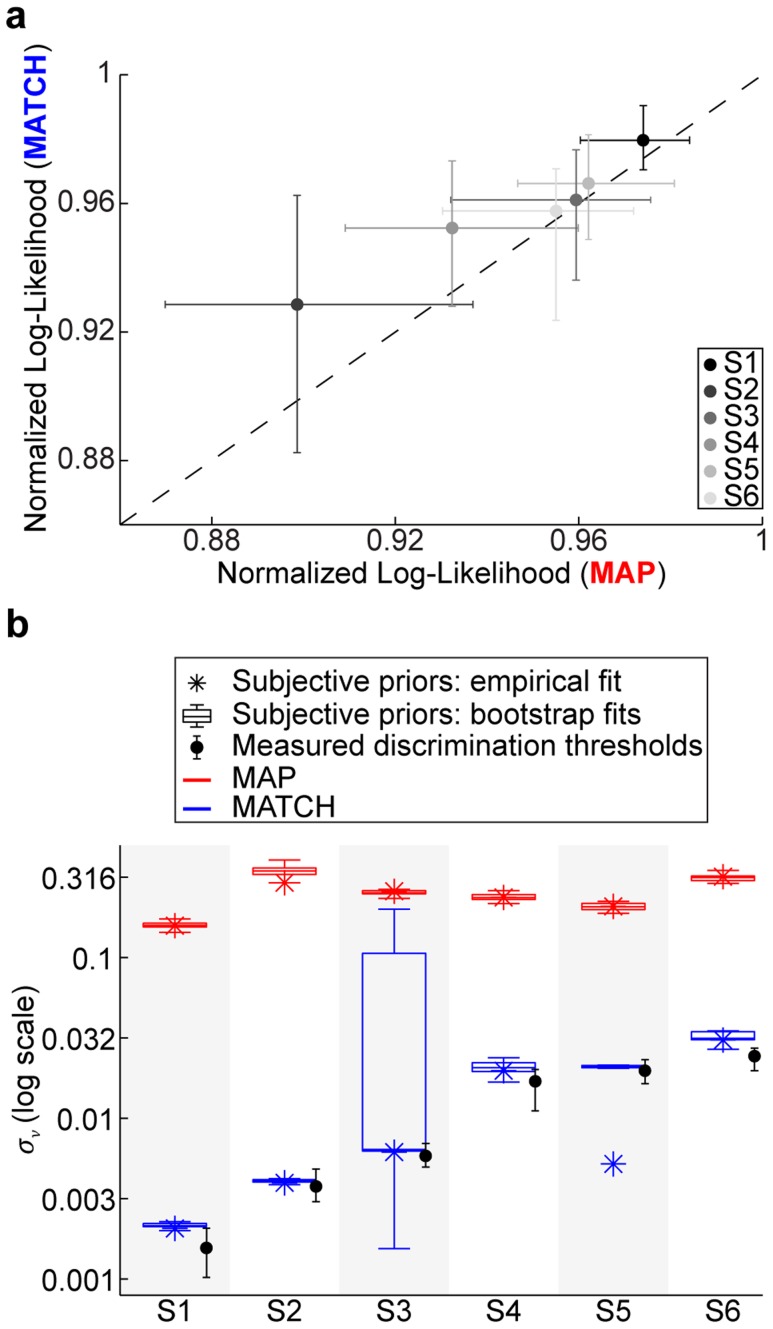
Likelihood comparisons for model fits. (a) Normalized log-likelihoods (see Figure 8) for MAP and MATCH. Data points denote median and bootstrapped 95% CIs. Dashed line depicts the unity line. (b) Boxplots depicting the range of the fitted sensory uncertainties (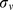) for MAP (red) and MATCH (blue). Stars denote fitted values to the measured data. Boxplots denote the median, 50%, and 95% CIs of the bootstrapped data. Black points denote measured discrimination thresholds for each subject and their 95% CIs.

When we included 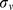 as a free parameter, both strategies accurately reflected individual subject's categorization behavior (fits not shown). However, we found that, without exception, MATCH behavior was still a better explanation of each subject's performance (Figure 13a). Moreover, in order for the MAP strategy to achieve this improvement in explanatory power, the sensory noise 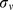 had to be *10–100* times larger than the measured values for each subject. In comparison, the fitted levels of 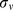 obtained from the MATCH fits were quite close to the individually measured discrimination thresholds for each subject (Figure 13b).

### Effects of noise on the categorical priors

Up to now, the model formulations assumed that subjects' estimates of the categorical priors were constant. However, this may not be true. Thus, we were interested in determining how trial-by-trial noise on the categorical priors may affect categorization performance. In particular, we wanted to test whether this additional noise could cause performance under an optimal decision strategy (MAP) to appear sub-optimal (MATCH).

**Figure 14:**
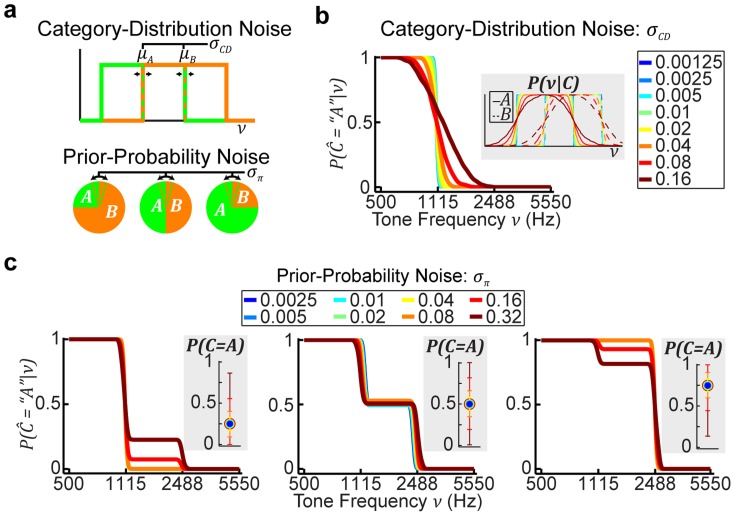
Simulations of behavior under the assumption of additional sources of categorical-prior noise. (a) Illustration of the two types of added noise: noise in the means of the category distributions (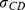, top) and noise in the category prior probabilities (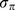, bottom). For the simulations, we computed the net effect on the psychometric function from 600 iterations of varying either the category means (b) or the category prior probabilities (c) assuming one of eight different levels of Gaussian noise. For (b) and (c), we also note the net effect on the corresponding estimates of the category distributions and category prior probabilities, respectively. (b) Net effects of noise in the category means (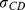) on the psychometric function for 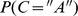 = 0.25. Colors denote the level of added noise. The effects were similar for each prior probability. (c) Effects of prior-probability noise (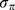) on the psychometric function. Panels depict effects for 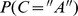 = 0.25, 0.5, and 0.75, respectively. Insets for each panel depict mean and 95% CIs for 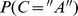. Colors denote level of 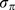 noise. Note, because probabilities range from 0 to 1, samples were fixed to remain within the range 0–1.

We conducted a series of simulations in which we added noise to both the means of the category distributions and the prior probabilities (Figure 14a). Increasing category-distribution noise (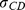) led to decreases in the slope of the psychometric function (Figure 14b). Note, even though the net effect of this noise is similar to having constant Gaussian-shaped distributions (Figure 14b, inset), the predicted categorization performance is different from the MAP predictions with constant Gaussian-shaped distributions (see Figure 6). In the latter case, there is no effect on the slopes of the psychometric function.

Increasing prior-probability noise (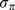) exhibited qualitatively different effects on performance as a function of 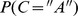 (Figure 14c). First, under asymmetric prior-probability conditions (i.e., 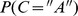 = 0.25 or 0.75), sufficiently small levels of 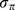 (e.g., below ∼0.08) did not substantially influence the psychometric function (Figure 14c, left and right panels). However, larger levels of 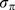 caused the function to exhibit plateaus. Moreover, depending on the level of 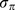, we could observe over-, under-, or true probability matching; compare the bright and dark red traces in the left and right panels of Figure 14c. Interestingly, when the prior probabilities were symmetric (i.e., 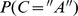 = 0.5), *any* level of 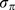 led to psychometric functions with a characteristic plateau.

**Figure 15:**
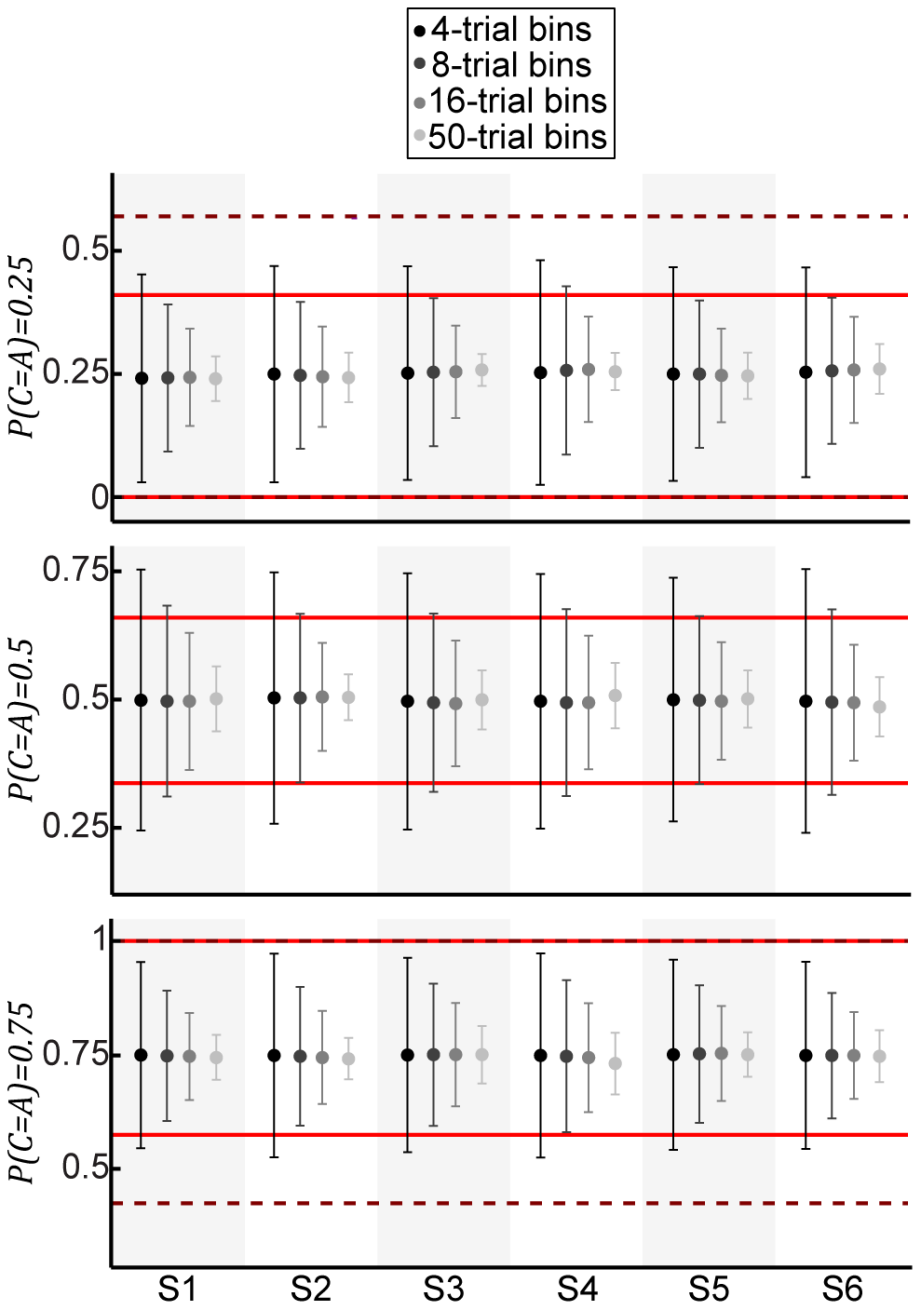
Variability in category prior probabilities computed as running estimates of 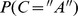 over different lengths of stimulus history (i.e., number of trials). Panels depict mean ± 1 SD of running averages of the stimulus history in the experiment for 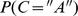 = 0.25, 0.5, and 0.75, respectively. Shading of data points denote different bin lengths. For comparison, the true 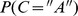 estimates ± 1 SD of the largest 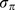 noise levels from Figure 14 are depicted with solid and dashed lines. Colors of lines are the same as in Figure 14c.

One potential interpretation of this noise is that subjects' categorical priors are non-stationary. Specifically, we hypothesized that subjects estimated the categorical priors only over recent trial history. To investigate this hypothesis, we computed running estimates of 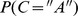 over different bin lengths of consecutive trials and compared the variability in these estimates with the levels of 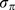 that yielded step-like psychometric functions. We found that the variability in 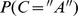 over relatively short bin lengths (i.e., generally <16 trials) was generally consistent with these 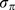 levels (Figure 15).

## Discussion

We found that subjects learned the categorization task to varying degrees. All but one subject could use the category-prior information to solve the task. Subjects learned general characteristics of the category distributions (i.e., high versus low frequencies) and the category prior probabilities as early as the first session. This is consistent with previous work showing that the largest effects of category learning occur early in training and then are fine-tuned with further experience [34], [35]. Our finding that subjects learned the category prior probabilities is consistent with previous visual categorization tasks [5], [8], [9], [36]–[38]. However, the systematic evaluation of prior probabilities and category learning in this study is novel for audition.

One goal of this study was to test whether subjects employed an optimal decision strategy to perform auditory categorization under categorical ambiguity. In order to do this, we developed a single generative Bayesian model that allowed us to both predict and fit each subject's psychometric curve for all tested conditions under instances of either optimal or sub-optimal categorization behavior. A critical component of this approach was that we separately estimated each subject's perceptual noise by measuring frequency-discrimination thresholds.

One finding of our model predictions was that subjects' performances were not accurately predicted assuming the objective priors (i.e., box-shaped distributions). This suggests that subjects were limited in their ability to learn the objective priors. Indeed, our model fits were consistent with the hypothesis that subjects learned smooth approximations of the box-shaped distributions. This finding may not be surprising: previous work has demonstrated that subjects often assume approximate versions of experimental distributions when learning new behavioral tasks [39]–[42]. It is possible that the large degree of uniform overlap between the categories contributed to subjects' difficulties in estimating the category distributions. However, other evidence suggests that subjects can, to an extent, learn category distributions that are non-Gaussian [41], [43], [44]. Therefore, with extensive training, subjects might have been able to learn the objective priors.

Another important finding was that subjects' performances were more consistent with probability matching. This was the case after both predicting and fitting performance with our Bayesian model. Because this type of behavior reflects sub-optimal categorization, we conducted further analyses to investigate whether subjects actually implemented an optimal decision strategy but performed sub-optimally due to additional uncertainties [33], [45], [46].

Additional memory noise was unlikely to account for this possibility for two reasons. First, when sensory noise was a free parameter and could account for additional memory noise, probability matching still outperformed the optimal decision strategy. Second, the fitted values of the sensory noise for the optimal strategy were 10–100 times larger than our measured estimates (Figure 13). This difference between the measured and fitted values seems unreasonable given previous work on the effects of memory noise on frequency discrimination [31], [32].

We also simulated the effects of additional noise on the category distributions and prior probabilities. The results of the simulations suggested that a combination of category-distribution and prior-probability noise could lead to psychometric functions that mimic probability-matching behavior (i.e., shallow psychometric functions with a plateau), even though the decision strategy was optimal (see Figure 14).

Categorical-prior noise could reflect true uncertainty or subjects' tendencies to search for patterns in sequences of random events [23], [25], [47], [48]. One interpretation is that our subjects assumed that the categorical priors changed over time (i.e., they were non-stationary). Under this assumption, our analyses suggested that subjects' estimates of the categorical priors were reflections of the short-term stimulus history (see Figure 15). Future work is necessary to determine more quantitatively whether subjects whose performance is most sensitive to the local trial history are more likely to exhibit psychometric functions that mimic probability-matching behavior and how this effect changes after extensive training.

Together, our results suggest that the prevalence of probability matching in perceptual tasks might reflect model assumptions of stationarity that are not correct [7], [49]–[52]. In other words, the interpretation of subjects' categorical behavior should not focus on sub-optimal versus optimal decision strategies but, rather, should focus on the degree to which subjects assume the environment is stationary and which factors can impact these assumptions. For example, changes in cost-reward structures may not change subjects' decision strategy, but may influence their view of environmental stationarity [7], [8], [37], [50].
